# Spatial Analysis of Human Granulocytic Ehrlichiosis near Lyme, Connecticut

**DOI:** 10.3201/eid0809.020103

**Published:** 2002-09

**Authors:** Emma K. Chaput, James I. Meek, Robert Heimer

**Affiliations:** * Yale University**,** New Haven, Connecticut, USA

**Keywords:** ehrlichiosis, cluster analysis, geography, tick-borne diseases, statistics

## Abstract

Geographic information systems combined with methods of spatial analysis provide powerful new tools for understanding the epidemiology of diseases and for improving disease prevention and control. In this study, the spatial distribution of a newly recognized tick-borne disease, human granulocytic ehrlichiosis (HGE), was investigated for nonrandom patterns and clusters in an area known to be endemic for tick-borne diseases. Analysis of confirmed cases of HGE identified in 1997–2000 in a 12-town area around Lyme, Connecticut, showed that HGE infections are not distributed randomly. Smoothed HGE incidence was higher around the mouth of the Connecticut River and lower to the north and west. Cluster analysis identified one area of increased HGE risk (relative risk=1.8, p=0.001). This study demonstrates the utility of geographic information systems and spatial analysis to clarify the epidemiology of HGE.

 Historically, the study of the spread of diseases within populations has included a spatial component. New tools, including geographic information systems (GIS) and spatial statistics methods, enable epidemiologists to address the spatial aspects of disease rates and transmission more thoroughly and less subjectively. The emergence of tick-borne infections in the United States has been attributed to reforestation and second-growth forests, with the associated increases in reservoir and vector populations, as well as to human behavior changes including residential preferences and the increased popularity of outdoor recreational activities (1–3).

 Our study used a GIS and spatial statistics to analyze the spatial distribution of a newly recognized tick-borne disease, human granulocytic ehrlichiosis (HGE). This disease was first described in a series of patients from northern Minnesota and Wisconsin in 1994 (4). The agent of HGE^1^ “is most closely related to *Ehrlichia phagocytophila*, which infects sheep and cattle, and *E. equi*, which causes disease in horses. Recent research has suggested that rather than three separate species, these organisms are three variants of the same species (5–7). In the eastern and midwestern United States, the agent of HGE is transmitted to humans by the tick vector, *Ixodes scapularis*. This tick is also the vector of *Borrelia burgdorferi* and *Babesia microti*, the agents of Lyme disease and human babesiosis, respectively (8). The HGE agent is well established in vector populations in the Northeast (9–11), and infection with multiple *I. scapularis–*borne pathogens has been documented in both humans and wild mammal reservoirs (9,11–18). Since 1995, HGE has been a physician- and laboratory-reportable condition in Connecticut. In addition, an active surveillance system for HGE was established in 1997 in a 12-town area around Lyme, Connecticut (Figure 1), where Lyme disease was first described and remains highly endemic (19). This region has a total population of 83,600 and encompasses 330.7 square miles. During the 4 years of surveillance (1997–2000), the average annual incidence of confirmed cases of HGE in the 12-town area was 42 cases per 100,000 persons. For the same 4-year period, the average annual incidence of Lyme disease in the 12-town area was 234 cases per 100,000 persons.

**Figure 1:**
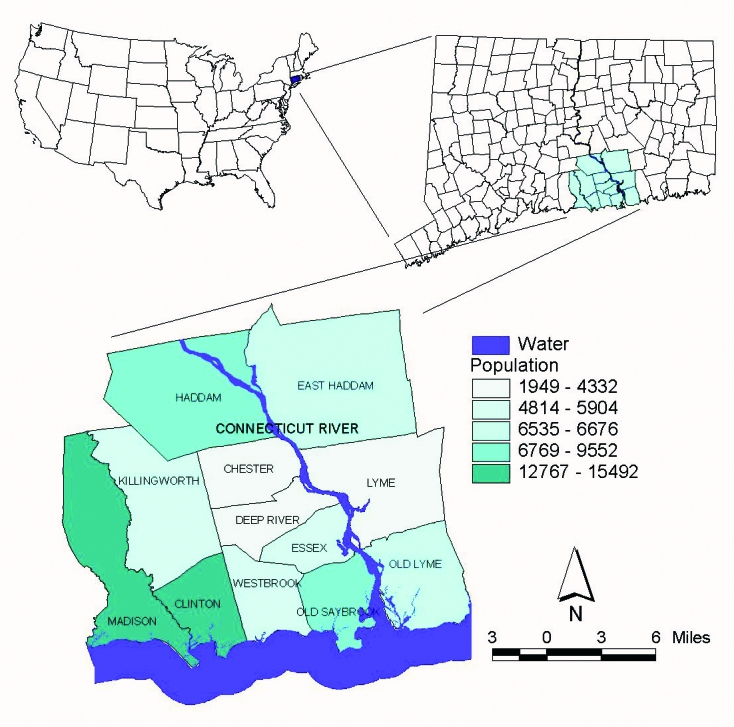
Surveillance area: 12-town area around Lyme, Connecticut.

 The use of a GIS with spatial statistics, including spatial filtering (smoothing) and cluster analysis, has been applied to other diseases, in which it is often used to analyze and more clearly display the spatial patterns of disease (20–25). Smoothing decreases the random variation associated with small case numbers and small populations, enabling disease gradients or holes to be observed that may not be apparent with raw data (20,26,27). Cluster analysis identifies whether geographically grouped cases of disease can be explained by chance or are statistically significant (23,28); it detects true clusters of disease from cases grouped around population centers (29). While many risk factors and environmental cues may be similar for Lyme disease and HGE, investigating the spatial nature of the latter in an area known to be endemic for both may increase our understanding of the epidemiology of HGE and enhance our ability to focus education and control efforts to reduce human disease risk. The goal of our study was to describe the spatial distribution of HGE within a highly endemic area and to provide the groundwork for further study to identify the environmental and landscape characteristics associated with increased risk for HGE infections.

## Materials and Methods

### Cases

 The confirmed cases of HGE analyzed in this study were identified through active and passive surveillance systems described elsewhere (19). Informed consent for participation in the active tick-borne disease surveillance study was obtained from all participants or their parents or guardians, according to a protocol approved by the Yale School of Medicine Human Investigation Committee. That committee approved a waiver of consent for this analysis. Only cases detected in 1997–2000 in residents of the 12-town area around Lyme, Connecticut, were included in the analysis. A confirmed case was defined as illness in a patient who had a seroconversion or ≥4-fold change in antibody titer between acute- and convalescent-phase serum specimens (by indirect fluorescent antibody or enzyme-linked immunosorbent assay), a positive polymerase chain reaction assay with primer pairs directed to genomic sequences specific to HGE, or detection of the specific 44-kDa protein band by Western blot analysis. A probable case was defined as an illness in a patient with a positive antibody titer from only a single serum sample or a <4-fold change in antibody titer between acute- and convalescent-phase serum samples.

### Geocoding Cases

 The home address was mapped for each confirmed case of HGE. We used home addresses based on the assumption that most people become infected with HGE near their homes. While peridomestic transmission has been established for Lyme disease (30), this assumption has not yet been tested for HGE. U.S. Bureau of the Census Topologically Integrated Geographic Encoding and Referencing system (TIGER/Line) maps, which contain street segments and address ranges for the 12 towns, were obtained from the University of Connecticut Map and Geographic Information Center (MAGIC). By using geographic information system software, ArcView GIS version 3.1 (Environmental Systems Research Institute, Inc., Redlands, CA), we geocoded home addresses to individual points in a new map layer by using the TIGER/Line street data files. Addresses were matched by town to decrease the error associated with similar names in different towns. An interactive matching process was used to increase the likelihood of achieving a match for an address. In addition, some addresses were identified by street maps and then manually added as points to the map. Town boundary and population census block group maps for each town were also obtained from MAGIC and were included as themes (map layers) with the geocoded points of cases. Once cases were geocoded, they were sorted by town and population census block group. Raw annualized incidence rates were calculated by using 1990 census data.

### Spatial Filtering (Smoothing)

 The technique of incorporating data from surrounding areas in an image or map to define a new data value for the area of interest is called spatial filtering. Spatial filtering can involve smoothing or sharpening the data of interest. To reduce random noise in the data that comes from the high variance characteristic of small populations or small case numbers (26), we performed the smoothing type of spatial filtering. Data were exported from ArcView, and the smoothing was done in the SAS statistical analysis software package, versions 6.12 and 8.0 (SAS Institute, Inc., Cary, NC). To decrease the variance to an acceptable level, a minimum “filter” number of 10 cases per area was established to calculate the disease rate. If an area (census block group) did not meet that minimum, smoothing was performed. This involved identifying a circle around the centroid of the census block group. The circle’s radius was enlarged until it included the centroid of the next closest census block group (based on the distances between centroids), and the number of cases was recalculated. This process was continued until the total number of cases circumscribed by the circle was >10. Then the disease rate was calculated individually for each census block group on the basis of this larger number of cases and larger population. Annualized incidence by census block group was calculated in ArcView after the data were exported back into the GIS. The technique of producing a smoothed map of disease rates allows for the display of data at a smaller geographic scale while preserving the stability of the estimated disease rates.

### Cluster Analysis

 Spatial cluster analysis was performed on the confirmed cases of HGE to test whether the cases were distributed randomly over space and, if not, to evaluate any identified spatial disease clusters for statistical significance (31). We applied the “spatial scan statistic” (31) to test the null hypothesis that the relative risk (RR) of HGE was the same between any block group, or collection of block groups, and the remaining block groups. By scanning varied size areas for possible disease clusters without prior assumptions of cluster size or location, we sought to avoid preselection bias (28). SaTScan software, version 2.1 (28), designed specifically to implement this test, imposed a circular window on the map. This window moved over the area and centered on the centroid of each census block group. The area within the circular window varied in size from zero to a maximum radius, never including >50% of the total population. The SaTScan software tested for possible clusters within the variable window around the centroid of each block group. Cluster analysis was performed with the default maximum spatial cluster size of <50% of the population and again with a smaller maximum cluster size of <25% to look for possible subclusters. For each window of varying position and size, the software tested the risk of HGE within and outside the window, with the null hypothesis of equal risk. This procedure compensated for the inherent bias in multiple testing (31).

 An additional cluster analysis was conducted by using both confirmed and probable HGE cases to address potential inclusion biases in the observed clustering of cases. These biases may have arisen because active surveillance cases were more likely to have provided both acute- and convalescent-phase samples than were cases detected through passive surveillance, and thus, had greater chance of being classified as confirmed cases and being included in analysis. Identical methods to those described above were used to perform the cluster analysis on the combined confirmed and probable cases.

## Results

**Figure 2:**
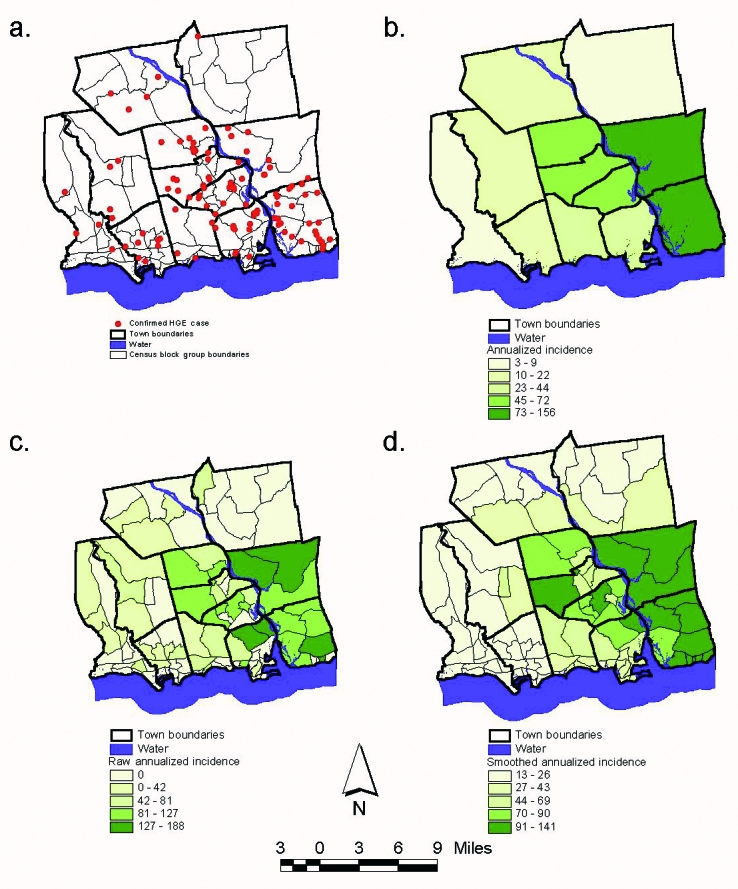
a. Confirmed human granulocytic ehrlichiosis (HGE) cases identified through active and passive surveillance systems, 1997–2000; b. Raw annualized incidence of confirmed HGE cases by town, 1997–2000*; c. Raw annualized incidence of confirmed HGE cases by census block group*; d. Smoothed annualized incidence of confirmed HGE cases by census block group.* *Cases per 100,000 persons.

 Two hundred forty-five cases of HGE were identified through the active and passive surveillance systems in 1997–2000. A total of 136 confirmed cases of HGE were identified; 128 (94%) of these were geocoded to points in an ArcView theme (Figure 2a). Addresses that were not geocoded consisted of two incomplete ones (street names with no number) and six post office boxes from Chester, Essex, Haddam, Lyme, Madison, and Westbrook.

 Annualized incidence rates for 1997–2000 were calculated by town and census block group by using 1990 census data to show the crude distribution of HGE in the 12-town area (Figure 2b,c). Rates by town ranged from 3/100,000 in East Haddam to 156/100,000 in Lyme. Rates by census block group ranged from 0/100,000 to 187/100,000 and demonstrated a high degree of random variation because of the small population size and low case numbers.

 Smoothing provided a clearer picture of the areas of increased risk on a smaller scale than by town. A filter number of 10 provided the most appropriate map of smoothed incidence rates (Figure 2d). This filter number decreased random variation and showed an increased risk of contracting HGE around the mouth of the Connecticut River with risk decreasing to the north and west.

**Figure 3:**
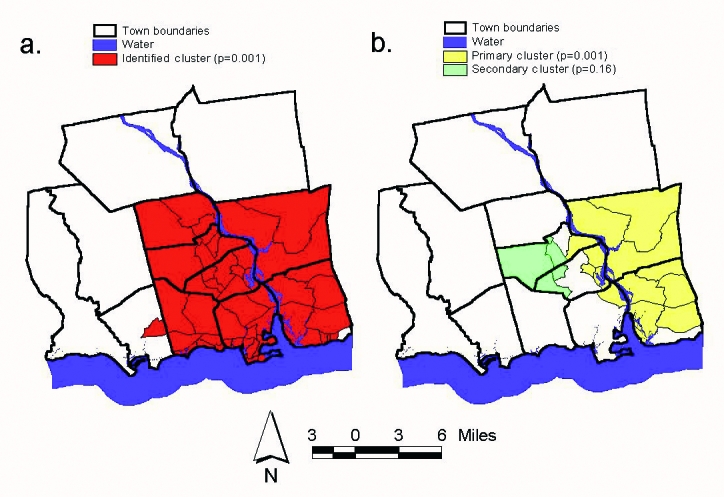
a. Single identified cluster of human granulocytic ehrlichiosis (HGE) cases within the 12-town area (maximum cluster size ≤50% total population), relative risk (RR)=1.8, p=0.001; b. Two identified clusters of HGE cases within the 12-town area (maximum cluster size ≤ 25% total population): primary cluster: RR=2.6, p=0.001, secondary cluster: RR=2.6, p=0.16.

 Using the maximum spatial cluster size of <50% of the total population, the spatial cluster analysis identified a single cluster that included all census block groups in the towns of Lyme, Old Saybrook, Chester, Essex, Deep River, and Westbrook, all but one in the town of Old Lyme, and one from the town of Clinton (Figure 3a). The identified cluster contained 46.1% of the area’s total population. The overall RR within the cluster was 1.8, with an observed number of cases of 106 compared with 59 expected cases. This elevated risk within a nonrandom pattern of disease distribution was significant (p=0.001).

 To investigate the possibility of smaller clusters, the same analysis was performed with a maximum spatial cluster size of <25% of the total population. Two clusters were identified, one including Lyme and Old Lyme as well as parts of Essex, Old Saybrook, and Deep River (Figure 3b). This cluster contained 18.2% of the total population and had an overall RR of 2.6 (p=0.001), with 61 cases observed compared with an expected 23 cases. The second subcluster included areas of Deep River and Essex (Figure 3b). This cluster contained 4.2% of the total population and had an overall RR of 2.6 (p=0.16), with 14 cases observed compared with 5 cases expected. While the primary cluster identified in this analysis was significant and showed a higher overall RR, the larger cluster from the first analysis, as a result of the lack of preselection bias, better represented the areas of increased risk for infection on the basis of the spatial distribution of HGE in the 12-town area.

 Cluster analyses were also performed on confirmed and probable case data. By using the <50% maximal cluster size, we included identical census block groups in the cluster (p=0.001). In the subcluster analysis that used the <25% cluster size, both subclusters identified with confirmed cases only were found to be statistically significant (p<0.005). The primary subcluster was geographically circumscribed when confirmed and probable cases were included in the analysis.

## Discussion

 Using a GIS and spatial statistics, we investigated the spatial distribution of confirmed cases of HGE and identified areas of increased risk within an area highly endemic for tick-borne diseases. Such diseases have become recognized as serious health threats in the northeast United States in the last 20 years because of increasing prevalence and heightened detection. Areas characterized by low residential density and a landscape of recently reforested deciduous forest are strongly associated with the risk for Lyme disease (30,32,33). Areas of high Lyme disease risk have been shown to also have an increased risk for HGE (19). Our study identified spatial variations in the risk for HGE in such an area. Furthermore, the analysis demonstrated that combining thorough surveillance information with spatial analysis techniques can increase understanding of the epidemiology of HGE within a highly disease-endemic area. The next step, to investigate the underlying causes of increased risk in the identified areas, will be analysis of landscape attributes and identification of the environmental variables characteristic of high-risk areas.

 The spatial statistics analyses clearly yielded a nonrandom distribution of HGE within the 12-town area. Spatial filtering (smoothing) identified areas of increased risk centered around the mouth of the Connecticut River, primarily on the eastern side of the river, in the towns of Lyme and Old Lyme. Increased likelihood of disease was seen on the western side of the river but was not as consistently high as the risk observed in Lyme and Old Lyme. Spatial cluster analysis identified a statistically significant cluster (RR=1.8, p=0.001) in the same area, around the mouth of the Connecticut River, including the towns of Chester, Deep River, Essex, Lyme, Old Lyme, Old Saybrook, and Westbrook. One census block group in southeastern Old Lyme was not included in the cluster, and one block group in Clinton was included. This cluster analysis was performed by using the default maximum spatial cluster size of <50% of the total population. Using this default method minimizes pre-selection bias of cluster size. However, to investigate the possibility of subclusters, additional cluster analysis based on a maximum spatial cluster size of <25% of the total population identified two subclusters, one significant (RR=2.6, p=0.001) and the second not significant (RR=2.6, p=0.19). The decrease in risk for HGE infection as one moves away from the coast is consistent with the results of Nicholson and Mather, who described a decreasing Lyme disease risk with increasing latitude in Rhode Island (34).

 The present study analyzed the associations between human population and human disease only. Gathering and including vector population data (including population density, distribution, and infection prevalence rates) and environmental variables in the risk analysis of HGE in the 12-town area may provide a more comprehensive view of the disease risk. The relationship between Lyme disease, *I. scapularis* vectors, and landscape characteristics has been studied from remotely sensed and field-gathered data (35–37), but it is unknown whether these relationships can be applied to other tick-borne diseases, including HGE. Increased Lyme disease risk has been well correlated with increased tick abundance and prevalence of infected ticks (34,35,38). The spatial distribution of Lyme disease rates is correlated with widespread tick populations and pathogen prevalence (25). Environmental risk factors and landscape characteristics associated with Lyme disease have been identified (22,35–37). Using techniques similar to those used for Lyme disease, combined with the results of this study, future research will include investigating the landscape characteristics associated with HGE. Further, discernment of the aspects of the natural history of HGE that are not understood, especially pertaining to the reservoir host, may supply additional information that can be used to further refine areas of HGE risk.

 While similar numbers of specimens were submitted for HGE testing to both the active and passive surveillance systems, the low rate of convalescent-phase specimen collection and the application of only one diagnostic test in passive surveillance resulted in fewer cases from passive surveillance being confirmed and included in the current analysis. Persons who live at the edges of the 12-town area may have been more likely to visit practitioners outside the active surveillance area. These case-patients would have been identified through passive surveillance but would have been less likely to be confirmed. While the practices participating in active surveillance were located throughout the 12-town area and include one practice outside that area, the lower rate of confirmed cases in the passive system may have biased the results toward the center of the surveillance area. However, the similar results obtained from the spatial statistics analysis that used both confirmed and probable cases suggest that this error may not have played a large role in the observed patterns of disease.

 Because of variations in testing throughout the 4 years of surveillance, analysis for temporal clusters was not possible. Retrospective testing of banked samples from previous surveillance years or continuing accumulation of surveillance data in years to come will be needed to investigate the temporal as well as spatial spread of HGE within the 12-town area. Temporal trends, combined with time series analysis of remotely sensed land cover and land-use data, may provide indications of future areas at increased risk for HGE. Concurrent analysis of the spatial and temporal distributions of other *I. scapularis–*borne diseases in this area, including Lyme disease and babesiosis, may clarify the similarities and differences in risk among these common vector-borne infections.

 Our study was based on the assumption that people acquire infection with the agent of HGE peridomestically, or near their homes. Falco and Fish (30) demonstrated that most cases of Lyme disease were acquired peridomestically, but no studies have investigated whether HGE infections are similarly acquired. While the life cycle similarities of these two pathogens support the assumption that HGE transmission dynamics are similar to those of Lyme disease, additional research is needed to test this hypothesis.

 This spatial analysis was limited to the described 12-town study area. This area of active surveillance was identified previously by its high rates of Lyme disease, and the towns to the east were excluded because at the time the study was initiated (1997) those towns had lower rates of Lyme disease compared with the 12 study towns. However, in this analysis, understanding of the spatial distribution of HGE would be enhanced if the towns to the east of the current study area were also included in the active surveillance, given the high rates of HGE in Lyme and Old Lyme. Stemming from this analysis, the towns to the east of the original study area (Salem and East Lyme) were added to the active surveillance area in 2002. The eastern limitation in our dataset highlights another use of spatial analysis and GIS. The tools and methods described in this study can identify areas where increased surveillance is recommended.

 Human behavior is a strong predictor of tick-borne disease risk, including how people move in their environment, their outdoor activities, and the individual protection they use to prevent tick bites. Reforestation in areas previously used for agriculture results in more favorable conditions for tick and reservoir hosts, while the trend towards residential preferences in well-shaded suburban and rural areas exacerbates the tick-human interactions. Change in human behavior concurrent with an ecologic transition further increases and alters tick-borne disease risk. Local weather variations and the periodicity of weather patterns also play a role in tick-borne disease risk. The combination of these factors results in a high variability of risk even within an area known to be hyperendemic for tick-borne diseases.

 On the basis of data on peridomestic Lyme disease infections (30), prevention strategies are recommended that focus on persons’ risk at home. In an area in which tick-borne diseases are highly endemic, aiming prevention strategies at areas of highest risk can potentially increase the program’s effectiveness. Persons at highest risk should be informed of that risk and of the possibilities for risk reduction. Funds spent on programs might be better spent on areas where cost-effectiveness can be maximized. At this time, practical prevention advice to prevent tick-borne disease in highly disease-endemic areas is elusive.

 The tools described in this article, GIS and spatial statistics, provide an opportunity to clarify and quantify the health burden from tick-borne disease within a highly endemic area and a foundation to pursue further investigation into the environmental factors resulting in increased disease burden. To implement specific and geographically appropriate risk-reduction programs, the use of such spatial analysis tools should become integral components in the epidemiologic description and risk assessment of tick-borne diseases.

## Footnotes

1Since this study was conducted, new nomenclature (*Anaplasma phagocytophila*) has been proposed; see Dumler JS, Barbet AF, Bekker CP, Dasch GA, Palmer GH, Ray SC, et al. Reorganization of genera in the families *Rickettsiaceae* and *Anaplasmataceae* in the order *Rickettsiales:* unification of some species of *Ehrlichia* with *Anaplasma*, *Cowdria* with *Ehrlichia,* and *Ehrlichia* with *Neorickettsia,* descriptions of six new species combinations and designation of *Ehrlichia equi* and ‘HGE agent’ as subjective synonyms of *Ehrlichia phagocytophila*. Int J Syst Evol Microbiol 2001;51:2145–65.
